# Cause and Prevention of Enteric (Typhoid) Fever

**Published:** 1898-06

**Authors:** J. G. Hirth

**Affiliations:** Milwaukee, Wis.; Professor of Hygiene and Assistant to the Chair of Internal Medicine, Milwaukee Medical College


### CAUSE AND PREVENTION OF ENTERIC (TYPHOID) FEVER.

BY. J. G. HIRTH, M.D., MILWAUKEE, WIS.

Professor of Hygiene and Assistant to the Chair of Internal Medicine, Milwaukee Medical College.

In order to fully appreciate the important relation that the cause, prevention and effect of typhoid fever bear to the commonwealth, it may be well to consider the prevalence and death rate of this disease at two different periods of time; and, for convenience of comparison, let me call your attention to the annual report of our American Health Bureau statistics relative thereto, for the years 1880 and that through which we have just passed—1895.

*1880 Census.*—In the census of 1880, there were reported 22,854 deaths from typhoid, to which at least 40 per cent. (9,141) should be added for inadequacy of reports, making a total of 31,995 deaths so reported. It is only just in this connection to note that this same report gives 20,231 deaths as due to malaria, and 12,640 deaths due to enteritis, making 32,871 deaths from both, to which should be added 40 per cent. of deaths reported, in order to approximate the actual number of deaths from these two causes, making 32,871; 40 per cent. of 32,871 =46,019 deaths, half of which (23,009) may be justly attributed to typhoid, making (31,995+23,009) 55,004 deaths from this one cause alone, which is a very conservative estimate.

*1895 Census.*—As regards the number of deaths from this one cause in the year just ended, we find that there are no fewer than 45,000 deaths reported from typhoid, to which should be added 30,000 estimated from previous reports resulting from typhoid, but credited to malaria and enteric disorders, making a very conservative estimate of 75,000 deaths from typhoid alone during the past year.

*Monetary Consideration.*—As regards this, we are safe to say that each life is worth $1,000, since the minimal cost of raising a child to the age of self-support is at least $1,000, which, however, is a low estimate. Since the vast majority of cases occur between the ages of 15 and 30, we may justly regard each loss of life thus sustained at $1,000, making a loss of $75,000,000 per annum. There are at least ten persons ill for every one that dies, so that the number of sick persons is 750,000; and with an average length of illness of four weeks this would make 21,000,000 days lost; each person must have an attendant, so multiply by two, making 42,000,000 days lost to to the State, each day of which is worth fifty cents, making a loss of $21,000,000; and to this add $1,000 for personal value of each death lost to the State, making $21,000,000+ $75,000,000, and we have $96,000,000 paid by the State as a tribute to criminal ignorance, carelessness and so forth, regarding this disease during the past year; and by the same process of reasoning, $70,000,000 for the year 1880, thus showing that the United States has done absolutely nothing to prevent the spread of typhoid fever.

*Cause.*—Notwithstanding the fact that a number of writers assume that the Eberth-Gaffky bacillus (1880) or Koch-Eberth bacillus is the specific germ of typhoid fever, among whom may be mentioned Osler, Wood, Fitz and Pepper among our American authors, aside from any German or English authorities, we must not fail to note that the four rules of Koch, particularly the crucial test of producing the disease in animal organisms by inoculation of a pure culture of the Eberth-Gaffky bacillus, has failed to produce the characteristic lesion.

*Koch's Rules Not Complied With.*—In addition to the fact that the four rules of Koch have not been complied with, we have much evidence that the essential lesion of so-called typhoid fever may be produced in animals by a number of micro-organisms other than the Eberth-Gaffky bacillus (Vaughan's "Leucomaines, Toxins and Antitoxins": Flugge and Vaughan); and pathologists have pretty generally agreed that the pathological findings of autopsies during the first few days of the so-called typhoid may differ both in lesion and bacteriological findings.

In 1890 Vaughan reported the isolation from water, supposed to have caused typhoid fever, a number of intoxicogenic germs, any of which proved noxious to rats, guinea pigs and dogs. The chemical poisons of two of these have been studied, and upon white rats these poisons produce symptoms which are identical with those which follow inoculation with living germs, and upon guinea pigs these chemical poisons proved fatal within twelve hours, with circulatory apparatus, kidneys, liver and spleen remaining sterile.

With Vaughan we have Klein, both of whom fail to agree that the Eberth-Gaffky bacillus is the sole cause of so-called typhoid fever, the former claiming that the Eberth-Gaffky bacillus as found in the spleen after death is an involution form of any one of a number of germs which are found in certain waters. (Vaughan's "Leucomaines, Toxins and Antitoxins," p. 208.)

*Varying Forms.*—In connection with this it may be well to note that Babes, in calling attention to the uncertainty of recognizing the bacillus of typhoid outside the body, has reported that he has distinguished nineteen different varieties of typhoid bacillus found in the bodies of typhoid autopsies. (*Zeitschrift fur Hygiene, Band* 9, p. 2.) Casedebat (*Annales de l'Institute Pasteur*, 1890, No. 10) has accurately described three different kinds of bacilli very much resembling typhoid, *e. g.*, bacilli found in Marseilles drinking water.

Infection through connection with typhoid patients is no more an open question. During the years 1861 to 1870 there were treated at the General Fever Hospital in London 3,555 cases of enteric fever, along with 5,144 patients not suffering from any specific fever, with the result that not one of the latter or the nurses contracted the disease. Dr. Gayler, in editing the third edition of the esteemed work of Murchison, remarks that subsequent experience down to 1882 of the London Fever Hospital is in complete accord with the above report. (*Enclycopædia of Hygiene*, Stevens and Murphy, Vol. II., p. 319.) In view of the above, and, indeed, I might cite innumerable cases, there can be no question but that enteric fever is not to be classed with the infectious diseases.

*Specificity.*—The fact that it is not at all infectious directly shows quite conclusively that the contagion is not a specific virus, and, furthermore, the virus contained in the excreta of patients suffering with the disease seems first to have to go through a period of incubation before it becomes active in infecting others with the disease, this period of incubation or evolution varying from three weeks to several months.

In view of the foregoing facts I do not believe that, after a careful study of the many various types of the so-called typhoid, taken in connection with the very meager specific bacterial evidence that we have thereto, especially since the germ is not invariably present, and since the bacterium coli commune of Escherich, which is a microbe present in the normal contents of the large intestines of the new-born and adult, being so similar in morphological, cultural and physiological respects to the Eberth-Gaffky bacilli that many observers cannot distinguish one from the other (Congress of Hygiene, 1891, Roux, Arloing and Rodet), we are justified in assuming that the varied class of symptoms, characteristically described by our authors as typhoid fever, are not due to one specific germ, but that it should be regarded as a mixed infection with one or more of several germs varying in degrees of specificity, acting as causal agents in producing this widely varying enteric disorder. If due to one germ, it must be one having a highly oscillating degree of virulency, and, theoretically, I think we are justified in assuming that the bacillus coli commune of Escherich when crossed with one or more of those germs found in polluted drinking water, is changed to a virulent germ, which elaborates a chemical poison (Toxalbumin) by feeding upon (*a*) the proteid present in the alimentary tract, thus serving to produce our mild forms of typhoid and so-called gastric fevers; (*b*) or when there is a high degree of susceptibility on the part of the patient or lessened constitutional resistance, the lymphoid tissue is the next area affected, Peyerian patches, mesenteric glands and spleen, as they are the points of least resistance, being the great cisterns of our filtered blood plasma, and a good nutrient media, thus serving to produce the varying forms of typhoid and kindred enteric disorders. "It has been shown by Roux and Yersen that the virulence of the pseudo-diphtheria bacillus can be raised by cultivating the bacillus with the streptococcus on a suitable nutrient material, or by passing through a series of animal organisms, with proper conditions of susceptibility, and so forth, that a virulence one hundred times greater in potency can thus be obtained." ("Diphtheria and the Use of Antitoxin in its Treatment and Prevention," by G. J. Hirth, *Milwaukee Medical Journal*, Vol. IV., No. 12, p. 398.) Here are two facts which may be influencing factors in the above, and may explain the varying gravity which we have all observed in typhoid.

*General Influencing Causes.*—General factors influencing the production of the virus of the disease bear an all-important relation to etiology, as also prevention, and we find that organic contamination, either vegetable or animal, of our natural waters, bears an important relation to the propagation or evolution of that class of germs commonly associated with enteric fever; and, in consequence, we find it most prevalent during that period of the year when the organic contamination of our water supplies, wells, cisterns ana river waters, is at its height—August, September, October and November in the temperate zones. And, on the contrary, when Nature's process of purification of our natural waters is at its height, we find it the least prevalent—winter, spring and early summer, though in thickly populated areas with defective drainage and sewage, it may be constantly present.

Another fact that organic contamination of our water supplies, either animal or vegetable, serves as the primitive predisposing factor in etiology, is shown that it prevails in India in its most virulent form during March, April and May, and during other seasons it is absent to the greatest extent, since in this area vegetable and organic contamination are at their zenith during February, March, April and May, thus giving time for the propagation of that class of germs associated with our enteric disorders, or probably to the evolution of the so-called Eberth-Gaffky bacillus, or a modified form of the same from other germs.

*Way of Entrance.*—The poison thus prepared gains access to the human system principally through drinking water directly or through drinking water infecting various foods; *e. g.*, in the recent epidemic at Stamford, Conn., 1895, there occurred 406 cases from April 15 to May 6, most of all having occurred in children and those who used the uncooked milk of a certain dealer, whose source of water for washing cans was a seap-well, located centrally between a number of cesspools and common privy vaults. Other examples of similar cases are on record in great number.

*Local Drainage and Sewerage.*—Infective drainage and sewerage as a source bears an undisputed relation to the propagation of the virus of enteric disorders, as practical experience shows that cities whose sewage and drainage are poor are invariably hot-beds of typhoid, even when the water dristribution is the very best in character; *e. g.*, the great hot-bed center for typhoid was at one time Munich, Bavaria, whose mortality from

1843 to 1849 was as high as 25 per cent. The drinking water at that time was gotten from wells in and around houses; waste materials of all kinds was collected in cellars; sewage was put in in 1860 with but little avail, and in 1868, pure water was piped many miles from springs and clear lakes, and to-day the average annual deaths were from 5 to 8 for 360,000 people, and these are imported cases. (Von Siemssen.) It is useless to say that with our modern improved plumbing that it is impossible for sewage, at its beginning, to infect a habitation unless there be a defect in same; at all times it is practically impossible to so construct a sewer, cesspool, privy vault, cistern or well that the contents of one will not be influenced by the other if in close proximity.

Irrespective of its source, a water which is a natural water, and taken from a river, unless it be from a mountainous district, is invariably unfit for water supply, owing to its contamination with vegetable organic matter; and, moreover, even though it be a still body of clear lake water, the source of water supply should never be taken from a point in close proximity to the entrance of sewers, or an area influenced through same. *E. g.*, Chicago has about twenty-four public sewers flowing into the lake, with innumerable private sewers, the result of which will invariably be either a direct contamination of the lake water with the virus of our enteric disorders or the overloading of the water with organic contamination, which serves as a culture medium for the same virus, either of which, or probably both, will serve as a source of infection, if the water is taken within an area so influenced, which doubtless comes within the limits of her present water supply, since she annually shows a high mortality, having reported 560 deaths during the year through which we have just passed, 1895, from this one cause alone. Again, Milwaukee furnished, we are happy to say, an admirable example of this great influence in having almost entirely stamped out this dreaded disease not by changing the natural source from which the water is taken, but by changing the location of the source from which the water was taken, thus eradicating the two above spoken of causes. The highly disastrous epidemic which prevailed in Duluth only a few years ago furnished another example, having reported 153 deaths in 1895. Hamburg, Germany, furnishes a most thorough practical application, in that even though the system of drainage and sewage is perfect, the water supply may be the only source of infection, and since their recent new system of filtration, still taking it from the same source, the mortality from this cause is practically *nil*.

Not only is it true that our public water supplies may become contaminated by sewage or even vegetable contamination, either constant or periodically, but there is no question to the mind of every rural practitioner but that these same causes which are acting in our cities are very frequently present and have their origin in farming communities, and that unclean barnyards, privy vaults, and especially water taken from streams collected from the vegetable areas of our alluvial soils, either of which or all may serve as foci of infection to our wells, poorly built cisterns and creeks, thus serving as a source of infection for our cases, having their origin *de novo* and in consequence a tributary source of infection for our larger bodies of natutal waters, our great rivers, which serve as a great sewer and, at the same time, water supply for nearly all cities located on their banks.

In connection herewith, my personal experience has furnished me ample data to prove the correctness of the foregoing statement, a short report of which may be of interest. Quincy, Illinois, a town of about 30,000 inhabitants, located high on the banks of the Mississippi, has constantly had much more than her share of typhoid deaths, ranging at about 1 per 1,000 population, according to her last annual reports; her system of sewage and drainage is certainly above par, and yet she invariably has a high autumnal mortality from typhoid with slight degrees of variation. The question may with justice be asked, Whence this source of infection? This can only be answered indirectly by saying that her source of water supply is the Mississippi, with no attempt whatever at filtration. Those of you who are familiar with her topographical geography know that on either side there is a wide belt of alluvial swamp soil, backed by high bluffs on either side, but particularly on the Illinois side. In this connection it may be said that during the autumn months this low area is most invariably the victim of a virulent malarial epidemic, though occasionally when the fall is dry the malaria may be entirely absent.

This Illinois bluff is backed by a rich, highland farming community; in this particular area, which is from three to ten miles north of the city, the surface water is collected by innumerable creeks which were dry during the most of July and August, but in the fall again became running streams. So constant was the prevalence of typhoid in one locality in this section during the months of September, October and November, that I have known as many as thirteen cases within an area of two square miles to have occurred in one fall, myself being counted among the number. It is of great interest to note in this connection that the victims of these epidemics were most invariably those who frequented this creek area of woods in pursuit of nuts, game, etc., since most every case was amongst males; thus showing conclusively that these cases had their origin doubtless *de novo* from this creek surface water, which became infected in the way spoken of elsewhere in this paper. The Mississippi river being more or less a collection of such waters, can it be wondered at that Quincy should at this coincident season of the year be the victim of the same allied disorders? I may also add that since this time (about twelve years ago), the borderland timber of this creek area, from one to two miles in width, has been cut down and the land put in cultivation, which has been the cause of rendering this small tributary quite extinct. Last fall when I visited this section I found that typhoid had entirely died out, and that the populace of this same area were the regular autumnal victims of malaria, which during my residence in this highland section some fifteen years ago was wholly unknown. This also shows that typhoid may arise independent of an antecedent cause, and that antecedent causes of a changeable character change the character of the enteric disorder, and in fact that they are conducive to such changes, seems highly probable; thus again showing that the virus of typhoid fever is not specific in character.

*Areas Most Influenced by Typhoid and Enteric Disorders.*—Statistically, it may be definitely proven that the cities located on the banks of our lowland rivers have a much higher death rate from typhoid and enteric disorders than those located inland or at the borders of our great lakes, and it cannot be questioned at this day but that pollution of a water supply by means of sewers—and indeed our great bodies of waters as regards rivers, come under this class—is responsible for the great majority of enteric disorders, including cholera, typhoid fever, dysentery, enteric diarrheal diseases and malarial fever.

*Prevention.*—Since there are no factors in the cause of typhoid so certainly established as the propagation and conveyance of the virus or antecedent by infected water, or by food contamination with infected water, by any of the many various ways, it necessarily follows that this source of contagion should receive the greatest amount of attention.

*Hamburg's Experience.*—A lesson of intense practical application may be learned from the experience which Hamburg, Germany, has undergone during the past several years. In the summer and fall of 1891 it was my privilege to observe practically, at the Hamberg Eppendorf Allegemeinen Krankenhaus—of about 2,300 beds—a large number of cases and autopsies of typhoid; in fact, several pavilions were given up to the care of these patients during the fall months, with a greater or less number during the remainder of the year. It may justly be stated that Hamburg's mortality from typhoid up until this time was almost a constant factor with only slight degrees of variation. In the fall of 1892 Hamburg became the victim of the cholera epidemic, a fact fresh in the minds of you all. Russian trading vessels from infected ports, unloading at the wharf, dumped their filth into the Elbe, which had never been anything more or less than a first-class sewer, notwithstanding the fact that the public water supply, without any attempt at filtration, was taken only a few blocks from the entrance of the public sewers. An immediate investigation into the prevailing sanitary conditions was of paramount necessity, as the number of deaths had increased within nine days to 484. The committee of investigation, consisting of Koch, the Public Board of Health Commissioner of Hamburg, and, I think, Pettenkofer, or Veit, reported that the existing source of infection was largely the public water supply; in consequence the water supply was cut off, special literature as regards the cooking of all foods and rules of hygiene being freely distributed; the result was that in twenty-eight days from the time that the first infection had reached its height (which was nine days after the first case was reported, the 30th day of August, 1892, having 1,081 cases reported with 484 deaths) it had almost entirely ceased.

In consequence of a specific sepsis (cholera) of the old system, which consisted of three great mud basins and sewers, it became necessary to construct a new system of water purification. That known as the solid sand-bath system, having its origin in England, was given favor, and within twelve months, by night and day work, her present system was completed. Some two years ago, while renewing my old associations at Hamburg, I happily found that such a thing as a typhoid patient was looked upon as a relic for purpose of study, and at that time when I called there was not a single case being cared for at the General Hospital.

*Description of Sand-Bath Filter.*—Even though the construction of this filter is known to many of you, a brief sketch of the mechanical structure may not be out of place. A large basin is dug into the surface of the earth, from one to four acres in area, about ten or twelve feet deep, bricked and cemented at bottom and sides, the latter being angular to about 45 degrees. A large drain is laid at the base by having prepared two cross trenches having one common outlet and being covered in entirely with large plates of stone, loosely placed. The base of the cement basin floor is covered with large boulder stones, successively graduated layers until small pebbles are reached (the depth being about four feet); on top of these very small pebbles, sand, which has been heated to red heat or or washed with sterilized water, is placed to a depth of three or four feet, and when packed is ready for use. These basins are flooded from beneath upward until the water reaches a level of three or four feet above the level of the sand, and the difference between the height of the level and the exit of the water will determine the rapidity of filtration, which is working most efficiently when filtering at the rate of from three to five inches per hour.

*Advantages of this Filter.*—(*a*) It removes all suspended matter present and at the same time organic matter plus germs which have become entangled in the upper film and layer of sands, which in time clogs the filter, and when the filter is cleaned, they are thus removed.

(*b*) It must not only be understood that sand acts best as a mechanical strainer, but physics has shown that finely divided solid substances such as sand exert the power of condensing and collecting oxygen, which serves the purpose of oxidizing organic matter held in solution, and possibly also the germs, since they are our simplest organic forms of life, endowed with little resistance. (The Eberth-Gaffky bacillus and bacilli closely allied are pretty generally accepted to be devoid of sporulation.) They also lose their integrity and diappear from water combined with soprophytic bacteria (Tysen), though in sterilized water they remain vital, according to Klemperer, for three months or more, are not destroyed by freezing, hence the ice supply is a factor which may account for sporadic cases or even epidemics, but to my mind this is not likely to occur, because at the time the ice is being frozen, Nature's processes of purification are at their height; again it may be true that even after a pure ice is cut it may be contaminated by water stirred up at the sides of a polluted stream. Please do not understand from what has been said that water polluted with sewage or vegetable contamination (albuminoid ammonia) is dangerous to life *per se*, but it is an indicator that such water will serve as a propagating culture medium and carrier for a variety of germs, including Koch's cholera bacillus, malaria bacillus, Eberth-Gaffky bacillus, the varying forms of bacteria which produce our summer diarrheas (Vaughan, Booker and Escherich). I do not think that diphtheria bacilli should be classed with this number, for clinical observations do not associate its etiology with polluted water supplies, but, like unto influenza, with poor ventilation.

*Organic Contamination Not Noxious Per Se.*—As regards organic contamination, *per se*, not being noxious to life, Emmerich, of Munich, has given the subject a practical test. For fourteen days he drank daily one liter of very filthy water, both chemically and physically impure (almost pure sewerage), with no deleterious effects; control experiments on others gave similar results; in fact, he claims that it served as a therapeutic agent in curing the gastric catarrh from which he was suffering at the time. (Wolffugel: "Wasserversorging," Pettenkofer, and Siemssen's "Handbuch der Hygiene," 1st abth. 2 heft, p. 97.) However this may be, our inborn instincts of sanitary conscience and cleanliness make it imperative that even though a pathogenic-germ-free sewerage should be taken with impunity, it should not be taken; our finer instincts demanding purity of food and water, which are rules dictated by Hippocrates 365 years before Christ.

Again, Berlin may be cited as a city whose mortality from typhoid fever is almost nil. I have witnessed from three to seven post-mortems daily for two semesters, taken from the Charite Hospital, and never saw a post-mortem of typhoid fever. During this same time I took a course in physical diagnosis at the clinic of Professor Paul Guttmann, at the Moabet General Hospital—about 1,700 beds—and during the entire time we did not have one case for clinical teaching. It is needless to say that hygienically, Berlin is the cleanest city in the world, as also to say that she has all water under bacteriological control, most modern sand filters, which have been recently put in at some distance up the Spree river (ten miles) at Muggle See, at the expense of the old sand-bath process which was condemned for the reason that it took its water from a point too near the city.

*Conclusions.*—In view of the foregoing we may conclude: (1) Typhoid fever occurs through taking into the body virus mixed in character, or, if specific, having widely oscillating degrees of virulency, which practically nullifies its specificity. (2) The virus or antecedent causes find access to the body always through the alimentary tract through the agency of water either directly or indirectly by drinking or eating. (3) That bad drainage, sewerage and vegetable contamination are the prime factors influencing water contamination. (4) That the portion of our large bodies of still waters (lakes) near the entrance of sewers, and our lowland rivers are so affected. (5) That water so infected or prepared for infection should never be used as a water supply unless (*a*) boiled or (*b*) filtered. (6) That when this process (sand filtration) is not practical it should only be taken from springs, subterranean streams, clear lakes or rivers not influenced by organic contamination. (7) That the filter having the requisite degree of practical efficiency (and the only adequate process of purification until the present), is the modern sand-bath filter, properly constructed and manipulated. (8) That all the rules of hygiene pertaining to the sick-room should be rigidly observed, and that both secretions and excretions of those ill of the disease should be disinfected by carbolic acid. (9) That a highland farming community and rural districts are in no wise exempt from this disease, and usually serve as the starting points for cases having their origin *de novo*. (10) And, lastly, that even though it may be necessary to wait until the future will have become the present in order to settle the question of specificity, the factors influencing the cause and prevention will always remain the same.—*Medical Times.*

